# Evaluation of peripheral cannulation technique among nurses in maternity and Dr. Jamal Ahmad Rashid pediatric teaching hospitals in Sulaimaniyah, Iraq

**DOI:** 10.1186/s12912-023-01349-y

**Published:** 2023-06-05

**Authors:** Gona Othman Faris, Awayi Ghazy Abdulkareem, Niyan Hakim Ismael, Delan Jamal Qader

**Affiliations:** 1 Department of Maternal Neonate Nursing, College of Nursing, University of Sulaimani, Sulaimaniyah, Iraq; 2 Department of Pediatric Nursing, College of Nursing, University of Sulaimani, Sulaimaniyah, Iraq; 3 Department of Adult Nursing, College of Nursing, University of Sulaimani, Sulaimaniyah, Iraq

**Keywords:** Peripheral cannulation, Technique evaluation, Nursing practice, Descriptive-analytical study

## Abstract

**Background:**

Obedience to the excellent standards of nursing practice is the ultimate attitude to develop patient outcomes and avoid nursing process related-infections. Inserting the peripheral intravenous cannula is the utmost mutual aggressive technique achieved in nursing care for patients. Therefore, nurses must have adequate knowledge and practice to ensure the procedure’s success.

**Objectives:**

To determine the peripheral cannulation technique evaluation among nurses working in the emergency departments.

**Methods:**

This descriptive-analytical study was conducted at Maternity and Pediatric Teaching Hospitals in Sulaimaniyah, Iraq on 101 randomly selected nurses, from 14th December 2021 to 16th March 2022. Data collection was carried out through a structured interview questionnaire aimed to gather the nurses’ general characteristics and an observational checklist to assess the nurses’ pre, during and post practices regarding peripheral cannulation technique.

**Results:**

According to general practice, 43.6% of nurses had an average level, 29.7% had a good level, and 26.7% had a poor level of practice in the evaluation of the peripheral cannulation technique. Our study also showed a positive association between socio-demographic characteristics of the studied samples with the overall level of practice regarding peripheral cannulation technique.

**Conclusions:**

Nurses were not practised peripheral cannulation technique appropriately; however, half of the nurses’ had an average level of practice, although their practices were not followed the standard protocols.

**Supplementary Information:**

The online version contains supplementary material available at 10.1186/s12912-023-01349-y.

## Introduction

Peripheral intravenous cannulation (PIC) is the commonly used intravenous process in medical practices [1]. However, it is an invasive procedure implemented mainly in hospitalized patients [2] to administer diverse solutions, medications, blood and its products. The technique is almost same in children and adults [3].

PIC is an essential part of an expert nursing exercise in healthcare practices for various dedications [4], which insert and remain for a limited duration of time mainly based on the patient’s situation with a possible danger of bacterial contaminations [2], which are mainly a part of nosocomial infections that related to increased hospitalization duration, as well as, rate of infection and death, with high cost [5].

Annually, intravenous medications are mainly given to most hospitalized patients (60%) using PIC worldwide, resulting in 6.2% of hospitals acquiring bacterial contamination and then septicemia [6]. Therefore, PIC is frequently related to localized than widespread infection. The most common complications of PIC are thrombophlebitis with a rate of 2.3–67%, and phlebitis in 1.5–60% of patients. However, complications such as infection, pain, leaking, dislodgement, extravasations and occlusions are not uncommon [7].

A common nursing action is an insertion, monitoring, and assessment of PIC sites as the cannula should be placed into the flexor aspect of the forearm, while the femoral vein is avoided as a high amount of normal flora are available in that region and might results in a high risk of infection [8].

Infection prevention is a potential element of a nurse’s profession. The majority of preventative strategies and interventions are included in standard nursing care. The nurse should be knowledgeable and familiar with the preparation and use of intravenous cannulas, as well as the avoidance, treatment, and management of local/systemic problems that can be supported by dynamic evidence-based practice guidelines [9].

The ability to implant and maintain medical devices is essential for all medical and nursing staff who works as medical personnel in hospitals and care for patients. Beginning with an intravenous remedy is one of the most effective ways as a combat medic that may avert mortality on the battlefield, including an intravenous cannula [10].

Intravenous cannulation is a common cause of nosocomial infections in hospitals due to bacterial flora migration on the site of introduction into the cannula’s cutaneous tract with the cannula’s outer surface. The use of superficial veins in the lower limbs is discouraged due to the increased risk of infection. If the cannula is inserted into the lower extremities, it might quickly become resistant [11]. Competent people should oversee the procedures that are being implemented. It is reasonable to anticipate recommendations to evaluate clinical practice for such a remarkable surgery when asked which catheter they preferred and why most nurses stated that they had formed their cannula insertion tradition from habit or experience accumulated over time or based on the availability of equipment in the hospital [12].

Based on the facts mentioned above, this study was assigned to evaluate the PIC technique among nurses concerning the care and conservation of intravenous cannula and difficulties they faced during work at Maternity and Dr. Jamal Ahmad Rashid Pediatric Teaching Hospitals in Sulaimaniyah, Iraq.

## Materials and methods

### Participants and study setting

This descriptive-analytical-observational research was conducted on 101 active nurses in Maternity Teaching Hospital and Dr. Jamal Ahmad Rashid Pediatric Teaching Hospital in Sulaimaniyah, Iraq, from 14th December 2021 to 16th March 2022, using a convenience sample technique.

### Measurement tools

Required data were obtained through direct interview and observational checklist [13] using a validated questionnaire that involves two parts. The first part had 7 items and was related to the nurses’ socio-demographic variables such as age, level of education, marital status, financial status, years of experience as a nurse, participation in training sessions concerning PIC technique, and duration of the training. The second part was an observational checklist modified by the researchers based on the guidelines for the PIC technique and divided into 3 sections with 37 items; the pre-practices, during practices, and post practices, each section had 11, 22, and 4 items, respectively and each question was answered by ‘Yes’ or ‘No’. The correct practice was marked as 1.0, while the wrong practice was scored as 0.0 (Supplementary file).

Meanwhile, the activity for each nurse performed during cannula insertion has been checked, while she/he performs her/his work, and each of them was observed on an individual basis without being informed during 3 visits for the same practice.

On the other hand, for pre-practice evaluation, a total score of 0 to 11 was used, when a score 0–5 was poor, 6–8 was average, and 9–11 was good practice. Total scores from 0 to 22 were used during-practices when score 0–10 indicated poor, score 11–16 showed average, and score 17–22 demonstrated good practice. Additionally, a total score from 0 to 4 was used to measure post-practices; when score 0–1 showed poor, score 2 represented average, and score 3–4 illustrated a good practice. The response was scored as ‘Yes ‘or ‘No’. The scales were classified as poor (≤49%) with scores 0–18, average (50–75%) with scores 19–27, and good levels (≥75%) with scores 28–37 for all practices. Individuals’ practice scores were calculated to give the total practice score (Table 1).

**Table 1:** Pre-, during, and post-practice score evaluation among nurses

Variable	Pre-practice	During-practice	Post-practice	All practice
	(1) For correct practice/(0) For incorrect practice			
**Total scores**	11	22	4.0	37
**Range**	0.0–11	0.0–22	0.0–4.0	0.0–37
**Poor (≤ 49%)**	0.0–5.0	0.0–10	0.0–1.0	0.0–18
**Average (50–75%)**	6.0–8.0	11–16	2.0	19–27
**Good (≥ 75%)**	9.0–11	17–22	3.0–4.0	28–37

#### Inclusion criteria

Active nurses who worked at the Emergency department of the hospitals regardless of age, gender, and nationality.

#### Exclusion criteria

Nurses who worked as a volunteer with less than 2 years of experience.

### Statistical analysis

Obtained data were analyzed using Statistical Package for Social Science (SPSS, version 24, Chicago, USA). Results were presented as frequencies and percentages. Chi-square test was used to determine the association between pre-practices, during, and post-practice with their socio-demographic data. Statistical difference considered significance (p˂0.05), highly significant (p < 0.001), and very highly significant (p < 0.000), while p > 0.05 was regarded as non-significant difference.

## Results

Regarding the nurse’s socio-demographic characteristics, 51 participants (50.5%) were aged 21–40 years, while nurses < 20 years old were 14 (13.9%). Furthermore, 53 (52.5%) nurses graduated from the institute, and only 12 (11.9%) had university certificates. Regarding marital status, only 55 (54.5%) were married, and 88 (87.1%) of the participants were from sufficient financial status backgrounds. Concerning work of experience in both hospitals as a nurse, 50 (49.5%) nurses had 6–15 years of experience in Maternity Teaching Hospital, and 58 (57.4%) participants had < 10 years’ experience in Pediatric Teaching Hospital. Also, 58(57.4%) nurses participated in training sessions concerning the PIC technique for 2–4 days (Table 2).

**Table 2:** Distribution of studied nurses according to socio-demographic characteristics

Socio-demographic characteristic	Frequency	%
**Age (Years)**		
≤ 20	14	13.9
21–40	51	50.5
> 40	36	35.6
**Level of education**		
Intermediate school graduate	14	13.9
Secondary school graduate	22	21.8
Institute graduate	53	52.5
College graduate	12	11.9
**Marital status**		
Married	55	54.5
Unmarried	43	42.6
Widow	3	3
**Financial Status**		
Sufficient	88	87.1
Barely Sufficient	12	11.9
Insufficient	1	1
**Experience in Maternity Teaching Hospital (Years)**		
˂6	32	31.7
6–15	50	49.5
> 15	19	18.8
**Experience in Pediatric Teaching Hospital (Years)**		
˂10	58	57.4
10–19	30	29.7
> 19	13	12.9
**Participation in training session concerning cannulation**		
Yes	43	42.6
No	58	57.4
**Total**	**101**	**100**

Concerning PIC technique evaluation, we found that 42.6% of the respondents had an average level, 30.7% had a poor level, and only 26.7% had a good level of practice regarding pre-practice. Moreover, 42.6% of the participants had an average level, 31.7% had a good level, and 25.7% had a poor level during practice. However, 80.2% of nurses had a poor level, 10.9% had an average level, and 8.9% had a good level of post-practice. Therefore, according to the overall practice, 43.6% had an average level, 29.7% had a good level, and 26.7% had a poor level of practice, respectively (Table 3).

**Table 3:** Distribution of information related to pre- during and post practices of nurses

Variable	Pre-practice Fr (%)	During-practice Fr (%)	Post-practice Fr (%)	All practices Fr (%)
**Poor**	31 (30.7)	26 (25.7)	81 (80.2)	27 (26.7)
**Average**	43 (42.6)	43 (42.6)	11 (10.9)	44 (43.6)
**Good**	27 (26.7)	32 (31.7)	9 (8.9)	30 (29.7)
**Total**	**101 (100)**	**101 (100)**	**101 (100)**	**100**

Fr: Frequency

In respect to determining the association between all practices of nurses and socio-demographic characteristics, there was a very highly significant difference between the level of all practice concerning PIC concerning age (p < 0.000), level of education (p < 0.000), years of experience in Maternity Teaching Hospital (p < 0.000), years of experience in Pediatric Teaching Hospital (p < 0.000) and participation in training session concerning PIC technique (p < 0.000) (Table 4).

**Table 4:** Association between practices concerning peripheral intravenous cannulation technique with socio-demographic characteristics

Socio-demographic	Poor Fr (%)	Average Fr (%)	Good Fr (%)	Total	p-value
**Age (Years)**					
≤ 20	12 (44.4)	2.0 (4.5)	0.0 (0.0)	14	< 0.000*
21–40	9.0 (33.3)	29 (65.9)	13 (43.3)	51	
> 40	6.0 (22.2)	13 (29.5)	17 (56.7)	36	
**Level of education (Nursing)**					
Intermediate school	12 (44.4)	1.0 (2.3)	1.0 (3.3)	14	< 0.000*
Secondary school	4.0 (14.8)	10 (22.7)	8.0 (26.7)	22	
Institute	7.0 (25.9)	32 (72.7)	14 (46.7)	53	
College	4.0 (14.8)	1.0 (2.3)	7.0 (23.3)	12	
**Experience in Maternity Teaching Hospital (Years)**					
< 6	18 (66.7)	11 (25)	3.0 (10)	32	< 0.000*
6–15	4.0 (14.8)	27 (61.4)	19 (63.3)	50	
> 15	5.0 (18.5)	6.0 (13.6)	8.0 (26.7)	19	
**Experience in Pediatric Teaching Hospital (Years)**					
< 10	21 (77.8)	25 (56.8)	12 (40)	58	< 0.000*
10–19	5.0 (18.5)	18 (40.9)	7.0 (23.3)	30	
> 19	1.0 (3.7)	1.0 (2.3)	11 (36.7)	13	
**Participation in training session concerning cannulation**					
Yes	20 (74.1)	18 (40.9)	5.0 (16.7)	43	< 0.000*
No	7.0 (25.9)	26 (59.1)	25 (83.3)	58	
**Total**	**27 (100.0)**	**44 (100.0)**	**30 (100.0)**	**101**	

*: Very highly significant difference using Chi-square test, Fr: Frequency

## Discussion

An intravenous cannula is one of the most routine procedures in hospitals. In several hospitals, intravenous cannula insertion is the primary job of nurses despite having specialized intravenous cannula teams in many centres to perform cannula insertion professionally [14]. Regardless of the regularity of this performance, few studies were conducted to guide the medical personnel to the most appropriate and successful means of conductance [15]. So, the present study evaluated the PIC technique among nurses at Maternity and Pediatric Teaching Hospitals in Sulaimaniyah, Iraq.

Analysis of socio-demographic characteristics of the nurses recruited in this study showed that 50.5% of nurses aged 21–40 years which is similar to other findings reported by other studies [10, 16]. Half of our study’s health care workers (52.5%) graduated from the nursing institute, while 11.9% of nurses graduated from the College of Nursing, nearly the same as another study that found 48.3% of nurses had graduated from the Nursing Institute [10].

Regarding their previous years of experience, 49.5% of nurses had 6–15 years of experience, and 57.4% had < 10 years’ experience in Maternity and Pediatric Teaching Hospitals, respectively. Opposite to our findings, Soliman et al., 2019 revealed that 49.4% of health care workers had < 3 years’ experience [17]. This study also illustrated that 57.4% of nurses reported not attending previous training courses concerning PIC technique which is agreed with another study that found 52% of nurses were not attended any training course [17]. However, it is opposite to Sriupayo et al. 2014, which found that > 50% of the studied nurses had previous training or conference attendance concerning PIC technique [18].

Furthermore, we found that 43.6% of nurses had an average level of practice, and 26.7% had a poor practice concerning PIC protocols, which might be related to that nurses were not practised appropriately. However, 29.7% of nurses’ level of practice about the PIC technique was good, but still, the practices were not according to the standard protocols. These outcomes were similar to those found in other studies that mentioned most nurses had unsatisfactory practice. Measures were generally higher during preparation, with a low level of practice during the actual performance and post-procedure of the PIC technique [10, 19].

Consequently, our results showed a very highly significant difference between all practices related to age, level of education, years of experience, and participation in training sessions concerning PIC technique with the level of practice. These findings did not agree with the findings of other studies that mentioned no association between socio-demographic characteristics of the studied nurses and the overall level of practice concerning PIC technique [17, 20]. However, Lund et al., 2012 found a positive association between level of practice with age, level of education, and participation in training courses [21]. These variations might be related to cultural factor, environmental factor, educational program/level, periodic nursing evaluation, and hospital facilities and services that should be offered to nurses continuously to improve their practical/clinical background without affecting their work quality.

## Conclusions

As revealed by the current study’s findings, nearly 1/2 of the studied nurses had an average level of practice, and > 1/4 had poor and good level practice in evaluating PIC technique. We also showed a positive association between the nurse’s socio-demographic characteristics and the overall level of practice concerning the PIC technique. Also, these results can assist in formulating an improve model to reduce the chances of PIC-correlated infections to raise the average care. Therefore, we recommend that hospital nurses receive thorough training in PIC techniques. These specially trained and qualified should instruct other hospital medical staff as documentation and labelling should be required during PIC procedures. In addition, nurses should know more about caring for and maintaining PIC as this procedure is necessary for any setting of health care. Hence, as part of health care providers, nurses should be aware of their practice level and how to handle the procedure in a very proper manner.

## Electronic supplementary material

Below is the link to the electronic supplementary material.

Supplementary Material 1

## Data Availability

The datasets used and/or analyzed during the current study available from the corresponding author on reasonable request.
