# Supplementary material for: Evaluation of peripheral cannulation technique among nurses in maternity and Dr. Jamal Ahmad Rashid pediatric teaching hospitals in Sulaimaniyah, Iraq
Source: BMC Nurs. 2023 Jun 5;22:188. doi: 10.1186/s12912-023-01349-y (PMC10240777; doi:10.1186/s12912-023-01349-y)
Supplement: Supplementary file 1 — Supplementary Material 1 [file 12912_2023_1349_MOESM1_ESM.docx]

ata analysis

Statistical analysis was performed using SPSS

Software version 16. Descriptive statistics was used

to analyze the data. Results were presented as

mean, frequencies and percentages. Chi square

was used to test for the association between

Data analysis

Statistical analysis was performed using SPSS

Software version 16. Descriptive statistics was used

to analyze the data. Results were presented as

mean, frequencies and percentages. Chi square

was used to test for the association between

Part two: Practice Cannula

1-Pre Procedure

Table (2): Distribution of Information related to pre-practices of nurses

| Level of practice | Total Score | Yes | No | **Variables : Pre Procedure of nurses** |
| --- | --- | --- | --- | --- |
|  |  | Fr.(%) | Fr.(%) |  |
| Good | 91 | 91(90.1) | 10(9.9) | None-sterile gloves |
| Good | 85 | 85(84.16) | 16(15.84) | Tourniquet or rubber band, warm wet towel or commercial heat pack |
| Poor | 49 | 49(48.51) | 52(51.49) | (3 ml) syringe filled with normal saline |
| Poor | 15 | 15(14.85) | 86(85.15) | Padded arm board(if immobilization of extremity is necessary) |
| Average | 72 | 72(71.29) | 29(28.71) | (1)Alcohol impregnate swab |
| Average | 69 | 69(68.32) | 32(31.68) | (1)appropriate suitable cannula |
| Good | 89 | 89(88.12) | 12(11.88) | (2)cotton wool balls |
| Good | 101 | 101(100.0) | 0(0.00) | tape (plaster) |
| Poor | 34 | 34(33.66) | 67(66.34) | (1) cotton bandage |
| Poor | 45 | 45(44.55) | 56(55.45) | Sharps Container |
| Poor | 19 | 19(18.81) | 82(81.19) | Isopropyl alcohol 70% solution hand solution |
| Note/ Sample(101) then the total score for each question has (101), Total scores in level of practices were graded assigning cut-off values used by (Poor practice : $\leq$ 49% (0-49) , Average practice : $50\%- \leq74$% (50-74), and good practice: ≥75% (75-101) | | | | |

Table 2 describes the responses of the participants to-

wards HB knowledge. Knowledge was assessed by ques-

tions focusing on HB etiology, sign and symptoms,

transmission, treatment and management. Each response

was scored as ‘yes’or ‘no’. The scoring range of the

questionnaire was 20 (maximum) to 0 (minimum). A cut

off level of ≤11 was considered as poor whereas > 11 was

considered as adequate knowledge about HB. Knowledge

scores for individuals were calculated and summed up to

give the total knowledge score

Table (2) describes the participants' responses to-wards the pre-practices of nurses. The practice was assessed by questions focusing on pre-practice that the assessment of Peripheral Cannulation Technique among nurses in Pediatric Teaching. Each response was scored as 'yes 'or 'no,' and the sample was 101. Individuals' practice scores were calculated. The scoring range of the questionnaire was 101 (maximum) to 0 (minimum). A cutoff level of 49% (0-49) was considered poor practice and a score 50%- 74% (50-74) was considered average practice whereas: ≥ 75 was considered good practice.

The highest score (101) was reported for tape (plaster) which was reported “Good practice”. Around 90.10% of the participants used Non-sterile gloves were reported as "Good practice," approximately (84.16%) of the nurses used a tourniquet or rubber band, warm wet towel, or commercial heat pack that was done "Good practice", about (88.21%) of the nurses had (2)cotton wool balls which was reported “good practice”. About (51.49% and 85.15%) has not (3 ml) syringe filled with normal saline and padded arm board by respectively which were reported "Poor practice". While, (28.71%) and (31.68%) of the nurses has not (1) Alcohol impregnate swab and (1) appropriate suitable cannula by respectively which was done by “average practice”. In addition, about (66.34%, 55.45, and 81.19%) of the participants did not make (1) cotton bandage, Sharps Container, and Isopropyl alcohol 70% solution hand solution which was reported as "Poor practice”.

2-during Procedure

Table (3): Distribution of Information related to during-practices of nurses

| Level of practice | Total Score | Yes | No | **During Procedure of nurses** |
| --- | --- | --- | --- | --- |
|  |  | Fr.(%) | Fr.(%) |  |
| Poor | 12 | 12(11.88) | 89(88.12) | Introduce yourself to the child parents |
| Poor | 31 | 31(30.69) | 70(69.31) | Explain what you are about to do clearly using appropriate language (if time and circumstances allow) |
| Good | 95 | 95(94.06) | 6(5.94) | prepare supplies and necessary equipment, cut tape in Xe cm for securing the IV catheter |
| Average | 66 | 66(65.35) | 35(34.65) | select appropriate cannula for child's sizes |
| Poor | 22 | 22(21.78) | 79(78.22) | procure assistance in restraining the child |
| Poor | 46 | 46(45.54) | 55(54.46) | wash hands |
| Good | 86 | 86(85.15) | 15(14.85) | put on non-sterile gloves |
| Good | 101 | 101(100.0) | 0(0.0) | choose IV set placement, apply tourniquet Tem above insertion sight (the most distal vein accessible should be selected first; this allows the use of more proximal sites if the initial attempt is unsuccessful) |
| Good | 98 | 98(97.03) | 3(2.97) | assess the vein visually palpation (if vein in apparent, have that patient contact and relax, local muscle repeatedly, apply a warm compress, or tap over the vein) |
| Average | 71 | 71(70.3) | 30(29.7) | Clean insertion site with alcohol wipes |
| Average | 56 | 56(55.45) | 45(44.55) | allow to dry insertion site after cleaning it. |
| Good | 76 | 76(75.25) | 25(24.75) | Grasp the area of distal to the site and pull slightly stabilize the skin and the vein. |
| Good | 91 | 91(90.1) | 10(9.9) | Avoid touching cannula tip or puncture site. |
| Good | 98 | 98(97.03) | 3(2.97) | Insert cannula by to degree, bevel up, almost parallel to skin |
| Good | 93 | 93(92.08) | 8(7.92) | Advance it until "flash" of blood is seen In hub by 30 |
| Good | 99 | 99(98.02) | 2(1.98) | advance the plastic catheter only by 10°, remove, needle, hold the cannula in and apply cover or saline filled syringe and apply pressure above insertion site with Index finger of non-dominant hand |
| Good | 99 | 99(98.02) | 2(1.98) | Remove tourniquet |
| Average | 55 | 55(54.46) | 46(45.54) | flush with 3 milliliters of normal saline to ensure patency |
| Good | 94 | 94(93.07) | 7(6.93) | secure the cannula |
| Good | 75 | 75(74.26) | 26(25.74) | ispose the needle in the sharp-container |
| Poor | 23 | 23(22.77) | 78(77.23) | document at the IV site date, size, Initials of person inserted it |
| Poor | 15 | 15(14.85) | 86(85.15) | Remove gloves and wash hands |
| Note/ Sample(101) then the total score for each question has (101), Total scores in level of practices were graded assigning cut-off values used by (Poor practice : $\leq$ 49% (0-49) , Average practice : $50\%- \leq74$% (50-74), and good practice: ≥75% (75-101) | | | | |

Table (2) describes the participants' responses to-wards the during-practices of nurses. The practice was assessed by questions focusing on during-practice that the assessment of Peripheral Cannulation Technique among nurses in Pediatric Teaching. Each response was scored as 'yes 'or 'no,' and the sample was 101. Individuals' practice scores were calculated. The scoring range of the questionnaire was 101 (maximum) to 0 (minimum). A cutoff level of 49% (0-49) was considered poor practice, a score 50%- 74% (50-74) was considered average practice whereas: ≥ 75 was considered good practice.

The highest score (101) was choose IV set placement, apply tourniquet Tem above insertion sight (the most distal vein accessible should be selected first; this allows the use of more proximal sites if the initial attempt is unsuccessful) which was reported “Good practice”. However, about (88.12%, 69.31%, 78.22%, 54.46%, 77.23% and 85.15) of the participants hadn’t (Introduce yourself to the child parents, Explain about to do clearly using appropriate language, procure assistance in restraining the child ,wash hands, document at the IV site date, size, Initials of person inserted and move gloves and wash hands) by respectively and which was done “poor practice”. Moreover, approximately (65.35%, 70.3%, 55.45% and 54,46%) of the nurses has selected appropriate cannula for child's sizes, to Clean insertion site with alcohol wipes, to allow to dry insertion site after cleaning it and flush with 3 milliliters of normal saline to ensure patency by respectively which was reported “average practice”. (75.25%) of the nurses used Grasp the area of distal to the site and pull slightly stabilize the skin and the vein and (90.1%) had Avoid touching cannula tip or puncture site which was reported “good practice”. In other hands, (97.03%, 92.08%, 98.02%, 98.02%, 93.07% and 74.26%) of the nurses has Insertd cannula by to degree, bevel up, almost parallel to skin, Advance it until "flash" of blood is seen In hub by 30, advanced the plastic catheter only by 10°, remove, needle, hold the cannula in and apply cover or saline filled syringe and apply pressure above insertion site with Index finger of non-dominant hand, Removed tourniquet, secure the cannula and ispose the needle in the sharp-container by respectively which was dene “good practice”.

3-Post Procedure

Table (4): Distribution of Information related to Post-practices of nurses

| Level of practice | Total Score | Yes | No | **Variables : Pre Procedure of nurses** |
| --- | --- | --- | --- | --- |
|  |  | Fr.(%) | Fr.(%) |  |
| Average | 74 | 74(73.27) | 27(26.73) | dispose the needle in the sharp –container |
| Poor | 21 | 21(20.79) | 80(79.21) | document at the IV site date , size , initials of person inserted it |
| Poor | 14 | 14(13.86) | 87(86.14) | Remove gloves and wash hands |
| Poor | 3 | 3(2.97 | 98(97.03) | Give the child/parents feedback |
| Note/ Sample(101) then the total score for each question has (101), Total scores in level of practices were graded assigning cut-off values used by (Poor practice : $\leq$ 49% (0-49) , Average practice : $50\%- \leq74$% (50-74), and good practice: ≥75% (75-101) | | | | |

Table (4) describes the participants' responses to-wards the Post-practices of nurses. The practice was assessed by questions focusing on Post-practice that assess the Peripheral Cannulation Technique among nurses in Pediatric Teaching. Each response was scored as 'yes 'or 'no,' and the sample was 101. Individuals' practice scores were calculated. The scoring range of the questionnaire was 101 (maximum) to 0 (minimum). A cutoff level of 49% (0-49) was considered poor practice and a score 50%- 74% (50-74) was considered average practice whereas: ≥ 75 was considered good practice.

The highest score (74) was reported dispose of the needle in the sharps –container, which was reported as “average practice.” About (79.21%, 86.14, and 97.03%) of the participants did not document at the IV site date, size, initials of the person inserted it, Remove gloves and wash hands and Give the child/parents feedback, which was reported as "Poor practice".
